# How ‘Drug Aware’ are our Glaucoma Patients?

**DOI:** 10.5005/jp-journals-10008-1181

**Published:** 2015-09-25

**Authors:** Chirayu Mohindroo, Parul Ichhpujani, Suresh Kumar

**Affiliations:** Final Year Student, Department of Ophthalmology, Glaucoma Service, Government Medical College and Hospital, Chandigarh, India; Assistant Professor, Department of Ophthalmology, Glaucoma Service, Government Medical College and Hospital, Chandigarh, India; Associate Professor, Department of Ophthalmology, Glaucoma Service, Government Medical College and Hospital, Chandigarh, India

**Keywords:** Eyedrops, Glaucoma, IOP.

## Abstract

**Background:** Poor knowledge, attitude and self-care practices (KAP) as regards medication compliance is a major concern in the management of glaucoma. This study aims to evaluate the knowledge, attitude regarding eyedrop instillation and self-care practices pertaining to eyedrops in diagnosed glaucoma patients.

**Methods:** In this cross-sectional, open-ended questionnaire-based study, 101 consecutive glaucoma patients on medication were recruited from an urban tertiary care hospital of North India. A self-designed 10-point KAP questionnaire that addressed patient-, medication-, environment- and physicians related factors was used.

For each desirable answer, the participant gives a score of 1 was given and for each undesirable answer a score of ‘0’ was given for each question. The total scores for each domain were calculated separately along with the total score. The association between the individual domain scores, the total score and various sociodemographic parameters were compared using unpaired t-test. Analysis of variance (ANOVA) test was used to compare the means, where the exposure variable had more than two categories.

**Results:** Out of 101 participants, 98% knew the reason why they were instilling the medicine. Only 61.4% subjects knew that the eyedrops should be stored in cool and dry place. Nearly 30% participants believed that two eyedrops could be instilled back to back. Half of the participants (55.4%) did not consider missing a dose of medicine to be significant. Majority (89.1%) of the participants asked the doctor about the drug dosage and timings and 71.3% of them did not use the eyedrops beyond 40 days after opening the vial. 37.6% participants believed that the medicine could be discontinued without asking the doctor, once the symptoms are relieved. Eighty percent patients checked the vial for correct drug name and expiry date before buying. 57.4% of the participants washed their hands before instilling the eyedrops. Only 23.8% patients asked their doctor for alternate medication name, in case they do not get the primary medication.

There were no statistically significant differences in the mean domain and total scores between males and females and between urban and rural patients.

There were no statistically significant differences in knowledge (p = 0.059) and attitude (p = 0.809) scores in people with different educational qualification. But education had a statistically significant relation with the practice scores (p = 0.004) and total scores (p = 0.047).

**Conclusion(s):** There exists marked variation in the reported practices, even in the very basic prerequisites of instilling eye-drops like washing of hands, checking the expiry date before the usage of eyedrops.

The findings in our study suggest a need to better educate our patients by providing them detailed information about eyedrop and its administration. This would help to reduce patients’ frustration, improve compliance and increase the efficacy of anti-glaucoma therapy.

**How to cite this article:** Mohindroo C, Ichhpujani P, Kumar S. How ‘Drug Aware’ are our Glaucoma Patients? J Curr Glaucoma Pract 2015;9(2):33-37.

## INTRODUCTION

Glaucoma is the leading cause of irreversible blindness worldwide.^1,2^ It is a group of ocular disorders with multifactorial etiology united by a clinically characteristic intraocular pressure-associated optic neuropathy and corresponding visual field defects and is said to be the third leading cause of blindness in India. When glaucoma is diagnosed, whether in the presence of normal or elevated IOP, it needs to be treated. Across the globe, topical medications are typically the first-line therapy, followed by laser and/or surgical therapy.

Eyedrops have been used in ophthalmology since ages. Despite many advances in management of ophthalmic diseases, ophthalmologists have not been able to find a way that obviates the eyedrop usage. Multiple clinical trials have shown that with effective medical treatment, vision loss can be prevented.^3-5^

Adherence, persistence and correct administration are three key elements for a successful topical pharmacotherapy for glaucoma patients. Because topically applied medications are the mainstay of glaucoma therapy, patients and family physicians need to be familiar with their stated purpose and their place in glaucoma therapy. As physicians, we all are aware that many of our patients do not use ocular hypotensives in the manner prescribed. Nonadherence in patients with glaucoma ranges from 5 to 80%.^6,7^ Reasons for this poor compliance are multi-factorial.

This simple open ended questionnaire-based pilot study aimed to check for the evaluation of the techniques being used by the patients for eyedrop instillations which can affect their drug compliance.

## MATERIALS AND METHODS

All the patients were thoroughly examined before inclusion in the study. The basic demographic details of each patient were recorded along with the type of glaucoma, name, number and duration of medications being instilled. All subjects were consented prior to enrolment and the protocol followed the tenets of the declaration of Helsinki. The KAP survey consisted of 10 questions and the main aim was to assess patients’ awareness and knowledge as regards the eyedrops. Data were analyzed using the SPSS version 21.00 (Chicago, Illinois, USA).

- *Study design:* Cross-sectional, questionnaire-based study
- *Study site:* Glaucoma service, ophthalmology outpatient department, Government Medical College and Hospital, Sector 32, Chandigarh.
- *Time period:* Two months
- *Sample size:* One hundred one consecutive patients of primary or secondary, open angle or angle-closure glaucoma, on medication, were examined.
- *Inclusion criterion:* All the diagnosed glaucoma patients self-administering topical antiglaucoma medication for at least 6 months.
- *Exclusion criterion:* Patients refusing consent for the study and patients who were debilitated and were taken care of by a caregiver. Patients suffering from Parkinson’s disease, Alzheimer’s disease and any other mental illness.
- *Pretesting methodology:* This questionnaire was pretested on 10 patients prior to starting the study. Following was kept in mind when designing the questionnaire and cross checked with the patients:
  – No negative questions were asked, therefore, none of the questions had a double negative answer.
  – The level of the questions was neither too difficult nor too unusual for the patient to understand.
  – Each question was unique; the same idea was not reflected in two questions.
  – Meaning and interpretation of question were clear.
  – Questions were simple, grammatically correct and free from technical jargon.

### Ethical Consideration

- Clearance from Institute Ethics Committee was obtained
- An informed consent was obtained from the patient prior to recruitment. The study did not involve the reviewing of clinical charts. The present study did not involve any invasive procedures. All therapeutic decisions were taken by the treating physician and no interference was done. Confidentiality of the patient was maintained and patient or his/her relative had the right to opt out of study at any given point of time.

## STATISTICAL ANALYSIS

IBM SPSS statistical software version 21 was used for statistical analysis. Sociodemographic variables like age and gender, residence, education, etc. were taken as explanatory parameters. Knowledge score, attitude score, practice score were taken as outcome variables. Descriptive analysis of all the explanatory and outcome parameters was done. All the categorical variables were presented in frequencies and percentages. The numerical variables presented in means and standard deviations. The association between explanatory and outcome parameters was assessed by calculating mean, mean difference and their 95% CI, f-static and p-value by independent T-test or ANOVA test. Graphical representation of analysis also presented in appropriate way.

(Citation: IBM Corp. Released 2012. IBM SPSS Statistics for Windows, Version 21.0. Armonk, NY: IBM Corp.).

## KNOWLEDGE, ATTITUDE AND PRACTICE QUESTIONNAIRE FOR EYEDROPS INSTILLATION

Cognitive debriefing was done to each participant in their native language.

Name:              Age/Sex:

Education:         Primary/Secondary/Tertiary/None

Diagnosis:         Residence: Urban/Rural

Duration since instilling drops: Number of topical drugs:

### Knowledge

**1.  Do you know the reason for which this medication has been given to you?**

      a.    Yes....................Ask the reason...............

      b.    No

**2.  Do you know where to store the medication?**

      a.    Cool and wet place (Baffle tray of refrigerator)

      b.    Cool and dry place (Refrigerator door)

      c.    Warm place (Over the refrigerator/Kitchen/ Bedroom).

**3.  Can you instill two eyedrops back to back with no interval?**

      a. Yes        b. No        c. Do not know

### Attitude

**4.  Does it matter if you miss a dose of prescribed eye drops?**

      a. Yes        b. No        c. Do not know

**5.  Do you ask your doctor about the drug dosage timings and how to divide the doses?**

      a. Yes        b. No

**6.Do you use a vial of eyedrops beyond 40 days after opening the vial?**

      a. Yes        b. No        c. Do not know

**7.  Do you agree that if you get relieved of the symptoms, then the eyedrop can be discontinued without a follow-up visit with your doctor.**

      a. Yes        b. No        c. Do not know

### Practice

**8.  Do you check the eyedrop vial for the correct drug name and expiry date before buying?**

      a. Yes        b. No        c. Trust the chemist

**9.  Do you wash your hands before instilling an eyedrop?**

      a. Yes        b. No        c. Sometimes

**10.  Do you ask for an alternative medication name from your doctor, in case the primary medication is not available?**

      a. Yes        b. No        c. Do not know

## RESULTS

A total of 101 participants were included in the study. The minimum age of the participants was 18 years, maximum age was 87 years with a mean age of 62.62 ± 15.48 years.

Out of total 101 participants, 53 (52.5%) were males. Eighty one (80.2%) belonged to Urban area. The number of people, who completed, the primary, secondary and tertiary levels of education was 19, 38 and 32 respectively while only 12 enrollees were illiterate.

### Knowledge

Nearly all patients, 99 (98%) knew the reason why they were instilling eyedrops. The proportion of people who expressed that, the eyedrops should be stored in cool and wet place was only 4%. Sixty two (61.4%) subjects said that the eyedrops should be stored in cool and dry place and the remaining 35 (34.6%) said the eyedrops should be stored in a warm place.

Twenty eight (27.7%) participants said that, two eye drops can be instilled back to back. One participant did not know about it and the remaining 72 (71.3%) knew that two eyedrops cannot be instilled back-to-back.

### Attitude

Fifty six (55.4%) of the participants believed that it mattered to miss a dose of medicine. Majority (89.1%) of the participants usually asked their doctor about the drug dosage and timings. Nearly 70% (71) of them did not use the eyedrops 40 days after opening the vial. Thirty eight (37.6%) of them thought that they could discontinue the medicine without asking the doctor, once the symptoms were relieved.

### Practice

Out of total 101 participants, 82 (81.2%) reported that, they check the vial for correct drug name and expiry date before buying. Fifty eight (57.4%) of the participants reported, they wash the hands before instilling eyedrops, 41 (40.6%) did not wash hands before instilling eyedrops and 2 (2%) people washed hands only sometimes. Twenty four (23.8%) people reported that, they ask their doctor for alternate medication name, in case they do not get the primary medication.

For each desirable answer the participant gave, a score of 1 was given and for each undesirable answer a score of ‘0’ was given for each question. The total scores for each domain were calculated separately along with the total score. The association between the individual domain scores, the total score and various sociodemographic parameters were compared using unpaired t-test. ANOVA test was used to compare the means, where the exposure variable had more than 2 categories.

There were no statistically significant differences in the mean domain and total scores between males and females (Table 1).

The residing area had no influence on the knowledge, attitude and practice scores, as there were no statistically significant differences between rural and urban population in the individual domain scores and the total score (Table 2).

There were very minor differences in knowledge scores in people with different educational qualification. These differences were statistically not significant (p-value 0.059) (Table 3).

**Table 1:** **Table 1:** Association of sex with different KAP scores (n = 101)

										*95% CI*			
*Parameter*		*Geider*		*Mean*		*Mean* *difference*		*p-value*		*Lower*		*Upper*	
Knowledge score		Male		2.34		0.06		0.611		–0.199		0.336	
		Female		2.27									
Attitude score		Male		2.92		0.34		0.062		–0.017		0.700	
		Female		2.58									
Practice score		Male		1.66		0.07		0.561		–0.185		0.339	
		Female		1.58									
Total score		Male		6.9245		0.48		0.122		–0.13303		1.10709	
		Female		6.4375									

**Table 2:** **Table 2:** Association of area of residence with different KAP scores (n = 101)

										*95% CI*			
*Parameter*		*Area of residence*		*Mean*		*Mean difference*		*p-value*		*Lower*		*Upper*	
Knowledge score		Urban		2.33		0.133		0.431		–0.201		0.468	
		Rural		2.20									
Attitude score		Urban		2.78		0.078		0.736		–0.379		0.535	
		Rural		2.70									
Practice score		Urban		1.72		0.466		0.004		0.150		0.782	
		Rural		1.25									
Total score		Urban		6.8272		0.67		0.086		–0.09766		1.451	
		Rural		6.1500									

**Table 3:** **Table 3:** Association of education with knowledge, attitude and practice scores (n = 101)

										*95% CI*			
*Parameter*		*Level of education*		*Mean*		*F-statistic (ANOVA)*		*p-value*		*Lower*		*Upper*	
Knowledge		Primary		2.05						1.80		2.31	
score		Secondary		2.21		2.560		0.059		1.96		2.47	
		Tertiary		2.47						2.26		2.67	
		None		2.58						2.16		3.01	
Attitude score		Primary		2.05						2.26		3.43	
		Secondary		2.21		0.323		0.809		2.39		2.93	
		Tertiary		2.47						2.43		3.13	
		None		2.58						2.59		3.24	
Practice score		Primary		1.21						1.01		1.41	
		Secondary		1.58		4.646		0.004		1.35		1.80	
		Tertiary		1.81						1.60		2.03	
		None		1.92						1.41		2.42	
Total score		Primary		6.1053						5.5512		6.6594	
		Secondary		6.4474		2.743		0.047		5.8822		7.0125	
		Tertiary		7.0625						6.4984		7.6266	
		None		7.4167						6.5405		8.2928	

There were no statistically significant differences in knowledge (p = 0.059) and attitude (p = 0.809) scores in people with different educational qualification. But education had a statistically significant relation with the practice scores (p = 0.004) and total scores (p = 0.047) (Table 3).

## DISCUSSION

The study group was heterogeneous in nature representing various sections of the community.

There can be little doubt that noncompliance, poor adherence and persistence with the prescribed topical medical management for glaucoma account for treatment failure. This study focuses on the same through a simple KAP survey. The current pilot study aims to address the insufficiency of data in the literature pertaining to many of the practical aspects of eyedrop administration. Although, most participants in this study reported little difficulty with the use, storage, and handling of eyedrops, the survey results demonstrate that broad variations in reported practices appear to exist.

Many commercial eyedrop preparations come with a broad range of storage requirements. Many drugs lose their effectiveness if stored in hot, moist, or brightly illumi-nated places. Heat, moisture, and light trigger irreversible physical changes to some of the chemical compounds in these prescription drugs.

Most drugs specify an upper range for temperature exposure during storage, 25°C (77°F), this temperature may easily exceed in areas such as the kitchen or bathroom or over the top of a refrigerator. Even the bedroom can have higher than these temperatures in warm climates without constantly maintained air conditioning. Some medications do not recommend storage temperatures in the near-freezing or refrigeration range. This may be especially problematic for the 4% who store medications in the refrigerator baffle tray, if instructions are not well understood or if the temperature in the refrigerator is not held constant.

Nearly 30% patients believed that two drops could be instilled back-to-back. Patients need to be aware of the ‘wash out effect’ due to back-to-back instillation of drugs. This may be because eyecare practitioners often prescribe eyedrops without properly explaining or showing the technique for correct instillation of eyedrops. This may occur because of the paucity of time in busy practice or lack of awareness of the fact that the patient may not be able to put the eyedrop correctly.

Approximately, 56% of the participants claimed to never miss a dose; these results are consistent with those of Stone et al,^8^ who in their study observed that 61% of the patients never missed a dose. Not missing a dose is an extremely important parameter for checking adherence to antiglaucoma instillations and is related with worsening of the disease.

In our study, 45 patients felt that missing a dose would not matter much. If patients missed a dose that implies that their drug vial lasts for more than 40 days. In addition, 26 (25.75%) patients used their drug vials beyond 40 days. Inappropriate dosing, because of either poor adherence or poor technique of instillation, means that patients are not receiving the prescribed medication as needed.

Thirty eight (37.6%) patients believed that if they got relieved of the symptoms, then the glaucoma medication could be discontinued without a follow-up visit with their doctor. This false belief can be detrimental to their disease status. This point should be addressed by each doctor while explaining the treatment to a glaucoma patient.

About 20% patients did not check the eyedrop vial for the correct drug name and expiry date before buying.

Nearly 75% did not ask for an alternative medication name from their doctor, in case the primary medication was not available. The possibility of a chemist substituting the prescribed drug for a wrong one cannot be ruled out. Hence, patients must ask for an alternative brand name for the prescribed drug from their doctor.

In our study, 58% of the participants reported washing of hands which was similar to the study done by Stone et al^8^ who reported that 61.9% subjects routinely washed their hands before instilling eyedrops. This was also consistent with a report from Tsai et al^9^ of 65% patients following the clean hands practice.

These results need to be interpreted with great caution. Firstly this is a pilot study and it mainly depends upon the patients’ self-report. Patients are known to overestimate their ability to comply with therapy.^10,11^ Secondly, enrolment in this study was limited to patients in glaucoma practices in either Chandigarh, Panchkula, Mohali and some parts of Punjab and Haryana. Greater geographical variation may yield varied outcomes. Furthermore, results may not be applicable to all forms of prescription or nonprescription eyedrop medications or to the therapies not specializing in glaucoma. Participants were asked about their use of antiglaucoma instillations only. Still, the results of this survey add to a growing body of literature that addresses many of the problems contributing to noncompliance with topical glaucoma therapy.^12^

## CONCLUSION

The aim of this study was to ascertain the knowledge, attit ude and practices followed by patients suffering from a chronic disease, such as glaucoma, which requires almost life-long topical medication therapy. An attempt was made to question about even the minute details associated with the use, storage and handling of eyedrops.

This study demonstrates that a broad variation exists in the reported practices, even in the very basic prerequisites of instilling eyedrops like washing of hands, checking the expiry date before the usage of eyedrops (which has been overlooked by many of the studies available in literature).

The findings in our study suggest a need to better educate our patients by providing them detailed information about eyedrop and its administration and also highlight many of the methodological problems. This would help to reduce patients‘ frustration, improve compliance, and increase the efficacy of antiglaucoma therapy.

## References

[1] Kingman S (2004). Glaucoma is second leading cause of blindness globally.. Bull World Health Organ.

[2] Resnikoff S, Pascolini D, Etya’ale D, Kocur I, Pararajasegaram R, Pokharel GP, Mariotti SP (2004). Global data on visual impairment in the year 2002. Bull World Health Organ.

[3] Lichter PR, Musch DC, Gillespie BW, Guire KE, Janz NK, Wren PA, Mills RP (2001). CIGTS Study Group. Interim clinical outcomes in the collaborative initial glaucoma treatment study comparing initial treatment randomized to medications or surgery.. Ophthalmology.

[4] (2000). The AGIS Investigators. The advanced glaucoma intervention study (AGIS):7: the relationship between control of intraocular pressure and visual field deterioration.. Am J Ophthalmol.

[5] Heijl A, Leske MC, Bengtsson B, Hyman L, Bengtsson B, Hussein M (2002). Early manifest glaucoma trial group. Reduction of intraocular pressure and glaucoma progression: results from the early manifest glaucoma trial.. Arch Ophthalmol.

[6] Nordstrom BL, Friedman DS, Mozaffari E, Quigley HA, Walker AM (2005). Persistence and adherence with topical glaucoma therapy.. Am J Ophthalmol.

[7] Zhou Z, Althin R, Sforzolini B, Dhawan R (2004). Persistency and treatment failure in newly diagnosed open angle glaucoma patients in the United Kingdom.. Br J Ophthalmol.

[8] Stone JL, Robin AL, Novack GD, Covert DW, Cagle GD (2009). An objective evaluation of eyedrop instillation in patients with glaucoma.. Arch Ophthalmol.

[9] Tsai JC, McClure CA, Ramos SE, Schlundt DG, Pichert JW (2003). Compliance barriers in glaucoma: a systematic classification.. J Glaucoma.

[10] Kass MA, Meltzer DW, Gordon M, Cooper D, Goldberg J (1986). Compliance with topical pilocarpine treatment.. Am J Ophthalmol.

[11] Kass MA, Gordon M, Meltzer DW (1986). Can ophthalmologists correctly identify patients defaulting from pilocarpine therapy?. Am J Ophthalmol.

[12] Zimmerman TJ, Zalta AH (1983). Facilitating patient compliance in glaucoma therapy.. Surv Ophthalmol.

